# Distinct breakfast patterns on satiety perception in individuals with weight excess

**DOI:** 10.1590/2359-3997000000133

**Published:** 2016-02-11

**Authors:** Aichah Ahmad El Orra, Milena Monfort Pires, Sandra Roberta G. Ferreira

**Affiliations:** 1 Departamento de Epidemiologia Faculdade de Saúde Pública USP São Paulo SP Brasil Departamento de Epidemiologia, Faculdade de Saúde Pública, Universidade de São Paulo (USP), São Paulo, SP, Brasil

**Keywords:** Satiety, dietary pattern, fatty acids, cardiometabolic risk

## Abstract

**Objectives:**

: Western dietary pattern predisposes to weight gain, insulin resistance and cardiometabolic diseases. Promoting satiety via modifications in diet composition could be useful to fight weight gain. Mediterranean diet which is recognized to be cardioprotective contains high fiber and unsaturated fat contents. We compared the effects of distinct breakfast patterns on satiety of individuals at cardiometabolic risk, and examined the correlation of satiety level after each breakfast intervention period with glucose parameters.

**Materials and methods:**

: In this 10-week cross-over clinical trial, 54 individuals with weight excess were submitted to 2 types of 4-week isocaloric breakfasts (2-week washout), one typically Brazilian and a modified one, differing concerning fiber and types of fatty acids contents. Clinical data were collected before and after each breakfast. A satiety scale was applied at fasting and 10, 30 and 120’ after breakfast consumption. Repeated measures ANOVA, Student t test or non-parametric correspondents were used; correlations were tested by Pearson or Spearman coefficients.

**Results:**

: Anthropometric variations after breakfasts were not significant. Only after the modified breakfast, reduction in blood pressure levels was observed. The satiety level did not show significant variation across each period or between the breakfasts. Non-significant correlation between satiety and glucose, insulin and HOMA-IR values after each intervention period was observed.

**Conclusion:**

: We conclude that different breakfast compositions do not alter satiety level, which is not correlated to glucose parameters in overweight individuals. Stronger modifications of daily meals might be necessary to differentiate satiety levels under distinct dietary patterns.

## INTRODUCTION

Habits of Western societies, including the Brazilian ones, have contributed to elevate the incidence of non-communicable chronic diseases, mostly due to the increase in body adiposity. As a consequence of current facilities for food consumption, individuals are consuming high-density high-fat and low-fiber foods, which characterize the typical Western pattern. Following the recognition of the deleterious impact of the high fat diet for metabolic disturbances that predispose to atherosclerosis (1,2), evidences related to the benefits of dietary habits of Mediterranean populations on cardiovascular mortality have emerged (3,4). Mediterranean dietary pattern is characterized by high content of monounsaturated fatty acids (MUFA), present in olive oil, and polyunsaturated fatty acids (PUFA), present in nuts and fish, as well as antioxidants minerals and polyphenols, present in vegetables, whole grains and wine. Considering the harms of saturated fat (SFA) and benefits of unsaturated fat on intracellular insulin signaling (5) and the results of dietary interventions (4,6), the scientific society has accepted the Mediterranean-style diet as indicated for cardioprotection (7,8).

Feeding-induced fullness is shown to be altered in obese individuals, and disturbances on the release and/or response to satietogenic substances following food intake were detected (9). How such disturbances contribute to weight gain is a matter of investigation. Satiety and hunger are regulated by multiple hormones (incretins, ghrelin, leptin, insulin, glucagon, somatostatin among others), which respond to nutrients present in the gastrointestinal tract that exert actions in hypothalamic neurons (10,11). Diet characteristics may influence hormonal and metabolic signals to central nervous system, favoring food intake and a positive energy balance. Studies in rodents have contributed to understand the mechanisms by which hormones inform the hypothalamus about the body adiposity level as well as the molecular pathways involved in neurotransmitters expression that modulates hunger and satiety (12). In clinical settings, comparisons of satiety level in response to distinct compositions of meals, for instance, one typically Western and another including Mediterranean components were scarcely investigated. It is possible that satiety handling could help the weight control and, consequently, result in cardiometabolic benefits (13).

In general, elevations of post-prandial plasma glucose induce satiety, although the magnitude of this effect varies according to the meal composition (14-16). A sharp rise in plasma glucose induced by simple carbohydrate consumption showed to be able to suppress satiety for a short duration, while the presence of fiber to prolong satiety (17). Despite evidence that dietary fat is more satietogenic than other nutrients, few data concerning differences in the perception according to its saturation grade of fatty acids are available (18). It is known that MUFA are more promptly oxidized than SFA, which could result in higher satietogenic power; however, in humans, studies are inconclusive (19).

Among the tools to assess satiety level reported by individuals following meal consumptions, the scale *Satiety Labeled Intensity Magnitude* – SLIM (20) has been proposed due to its simplicity, easy to use and sensitivity to quantify this perception in response to diverse composition of food intake. The SLIM shows good accuracy and enables better discrimination in comparison with others described (21).

Facing the perspective that knowledge on the impact of different dietary patterns in provoking satiety could contribute for the obesity control, this study aimed at comparing the satiety levels in response to two isocaloric breakfasts but with different compositions, one typically Western and one modified including Mediterranean components; also, at analyzing the correlation of satiety level after experimental periods of breakfast with plasma glucose, insulin and insulin resistance index.

## MATERIALS AND METHODS

### Sample

This study was approved by the School of Public Health Ethical Committee and conducted at the University of São Paulo Hospital. Individuals agreed and provided signed consent. The convenience sample was constituted by individuals aged 35 to 69 years, body mass index (BMI) ≥ 25 and < 40 kg/m^2^, and at least one additional abnormality: concentration of triglycerides ≥ 150 mg/dL or total cholesterol ≥ 230 mg/dL or LDL-c > 100 mg/dL or fasting plasma glucose between 100 and 124 mg/dL or systolic blood pressure ≥ 140 mmHg. Exclusion criteria were use of medications that influence hunger or satiety, psychiatric disorders, pregnancy and body weight variation > 5% during the last six months.

### Protocol

This was a 10-week cross-over clinical trial composed of two interventions in the breakfast, of 4-week duration each with a 2-week washout period between them. The breakfasts were isocaloric (energy content of 480 kcal), but differing according to the fatty acids composition and fiber content. The called “Brazilian breakfast” was prepared including: whole milk (180 mL), coffee (60 mL), sugar (10 g), French bread (50 g), butter (15 g) and mozzarella cheese (32 g). The “modified breakfast” was created including some components of the Mediterranean diet as following: skim milk (180 mL), coffee (60 mL), sugar (10 g), whole-grain French bread (50 g), ricotta cheese (40 g) with virgin olive oil (16 g) and peanuts (10 g). These preparations resulted in the same content of total fat but differing by the relative amounts of fatty acids. Participants were oriented to maintain their daily intake during the day, as well as their regular physical activities. They weekly received phone calls in order to reinforce compliance to the experimental protocol.

One week prior the start of study, as well as at the last week of consumption of each breakfast, two 24-hour food records and the satiety scale were collected. One food record was obtained face-to-face and the other by telephone, by a trained dietitian, using the multiple pass method. The standardization of equivalences of house measures of consumption (in grams) was performed based on a Brazilian-tailored directory of recipes and measures previously described (22,23). Dietary data were analyzed using the Virtual Nutri software. Energy intakes < 500 kcal or > 5,000 kcal/day were considered outliers and were excluded from analyses. Variables of interest for the present study were total energy intake (TEI), macronutrients, subtypes of fatty acids and total fiber intakes.

Level of satiety was assessed using the SLIM scale (20), in which the participant is asked to inform one of 11 conditions, distributed into a visual vertical line that ranges from “too full” until “too hungry”. To each condition in this scale (available at http://www.sciencedirect.com/science/article/pii/S0195666304001242) there is a correspondent numeric value (score of satiety). Negative values in the SLIM scale are indicative of hunger. Satiety data were obtained before and 10, 30 and 120 minutes after breakfast consumption.

Physical activity was evaluated using the short version of the International Physical Activity Questionnaire that was validated in Brazil (24), and was expressed in minutes per week.

At baseline and at the final of each intervention, anthropometric measurements, blood pressure and fasting blood samples were obtained for determinations of plasma glucose and insulin concentrations, employed for the calculation of insulin resistance index. Body mass index was obtained by the ratio of weight (in kilograms) to squared height (in meters). Waist circumference was taken in upright position during expiration, at midpoint between the last rib and iliac crest. Blood pressure was taken after resting for five minutes in sitting position with automated oscillometric device (Omron HEM-712C, Omron Health Care, USA), three times, being the mean of the last two measurement the final values of systolic and diastolic blood pressure levels.

### Analytical methods

Plasma glucose was determined by glucose oxidase method and insulin by enzyme-linked immunoenzymatic assay (AutoDelfia, Perkin Elmer Life Sciences Inc, Norton, OH, USA). These values were used to calculate HOMA-IR (*homeostasis model assessment of insulin resistance*), and used to estimate insulin resistance (25).

### Statistical analysis

Data were expressed as mean and standard deviation or error. Log-transformation was applied for non-Gaussian variables for analysis purposes, and values were reported as back-transformed into their original units. Changes in dietary and clinical data before and after breakfasts were compared by Student t test. The profiles of satiety score values across time after each breakfast were compared by repeated measures ANOVA. Correlations between variables were tested by Pearson or Spearman coefficients. P value < 0.05 was considered significant. Statistical analysis was performed using the SPSS version 17.0 for Windows.

## RESULTS

From 80 individuals, 54 completed both intervention periods of breakfasts. Among the dropouts there was a predominance of men. The frequency and doses of medications were maintained in the whole period, as well as the level of physical activity (data not shown). The sample was composed of 66% of women, with a mean age of 53.0 ± 1.3 years. As expected, participants had increased body mass index (30.8 ± 1.4 kg/m^2^). Table 1 shows that the mean values of waist circumference (105.2 ± 2.5 *versus* 98.2 ± 1.3 cm, p < 0.01) and blood pressure levels (138.6 ± 4.7 *versus* 123.5 ± 2.5 mmHg, p < 0.01) were higher in men, while HDL-c was higher in women (57.0 ± 2.3 *versus* 44.4 ± 2.0 mg/dL). Satiety scores before and after breakfasts did not differ between sex, except for higher values in women than men after 10 minutes of the modified breakfast.

**Table 1:** Clinical data and satiety scores of 54 participants according to sex. Values are expressed in mean and standard deviation

	Men n = 18	Women n = 36	p
**Clinical data**			

Age (years)	49.9 ± 2.4	54.6 ± 1.5	0.09
Body mass index (kg/m^2^)	31.6 ± 1.2	30.4 ± 0.8	0.32
Waist circumference (cm)	105.2 ± 2.5	98.2 ± 1.3	< 0.01
Systolic blood pressure (mmHg)	138.6 ± 4.7	123.5 ± 2.5	< 0.01
Diastolic blood pressure (mmHg)	80.4 ± 3.0	74.7 ± 1.6	0.07
Plasma glucose (mg/dL)	98.9 ± 2.6	93.8 ± 1.6	0.09
Total cholesterol (mg/dL)	202.4 ± 11.0	207.4 ± 5.8	0.67
HDL-c (mg/dL)	44.4 ± 2.0	57.0 ± 2.3	< 0.01
LDL-c (mg/dL)	121.4 ± 9.8	123.2 ± 4.2	0.84
Triglycerides (mg/dL)	179.5 ± 25.7	133.9 ± 9.6	0.05

**Satiety scores**			

Brazilian breakfast			
Fasting	-23.4 ± 5.8	-12.1 ± 3.7	0.10
10 minutes	73.3 ± 5.7	81.3 ± 2.9	0.17
30 minutes	54.2 ± 7.1	65.0 ± 19.6	0.12
120 minutes	23.7 ± 10.1	31.8 ± 5.9	0.50
Modified breakfast			
Fasting	-23.4 ± 5.8	-12.1 ± 3.7	0.10
10 minutes	69.2 ± 3.7	83.3 ± 2.8	0.04
30 minutes	57.7 ± 26.5	68.1 ± 3.5	0.11
120 minutes	14.9 ± 9.1	29.5 ± 38.5	0.20

**Figure 1:**
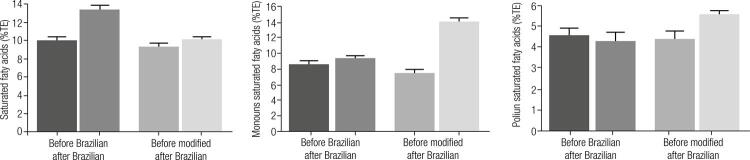
Changes in subtypes of fatty acids intake after intervention with Brazilian and modified breakfasts.

Dietary data at baseline and after Brazilian and modified breakfasts are shown in Table 2. Despite the orientation to maintain regular daily intake, increases in total energy (TEI) and fat intakes were verified in both interventions, which differed according to the distribution of types of fatty acids consumed (Figure 1).

**Table 2:** Mean values (± SEM) of intake of dietary variables before and after four weeks of Brazilian and modified breakfast

	Brazilian			Modified		
	
	Before	After	P-value	Before	After	P-value
Total energy intake (kcal/day)	1871 ± 66	1678 ± 83	0.01	1743 ± 81	1770 ± 48	0.75
Carbohydrate (% TEI)	52.0 ± 1.0	48.9 ± 0.9	0.03	54.3 ± 1.1	48.2 ± 0.7	< 0.01
Protein (% TEI)	15.5 ± 0.5	14.2 ± 3.2	0.05	15.4 ± 0.5	14.3 ± 0.4	0.04
Total fat (% TEI)	32.5 ± 0.9	36.9 ± 0.9	< 0.01	30.3 ± 0.9	37.5 ± 0.7	< 0.01
Saturated fatty acids (%TEI)	9.9 ± 0.4	13.4 ± 0.4	< 0.01	9.1 ± 0.4	10.4 ± 0.3	< 0.01
MUFA (% TEI)	8.7 ± 0.4	9.3 ± 0.3	0.23	7.7 ± 0.4	13.8 ± 0.4	< 0.01
PUFA (% TEI)	4.8 ± 0.3	4.5 ± 0.3	0.05	4.3 ± 0.3	5.6 ± 0.4	< 0.01
Total fiber intake (g/day)	12.5 ± 0.6	12.8 ± 0.7	0.73	12.9 ± 0.8	15.0 ± 0.7	0.04

TEI: total energy intake; MUFA: monounsaturated fatty acids; PUFA: polyunsaturated fatty acids.

As far as the Brazilian breakfast is concerned, in particular, the increase in TEI was due to higher SFA intake (27.9 ± 0.81 *versus* 20.8 ± 0.93 g after Brazilian and modified, respectively). Relatively to TEI, the consumption of SFA was elevated, in parallel with significant reductions in intakes of carbohydrates and proteins (p < 0.05)._._

After modified breakfast, elevation in total fat was observed which occurred mainly due to increased MUFA and PUFA intakes. Similar to the intervention with Brazilian breakfast, reductions in relative intakes of carbohydrates and proteins (p < 0.05) were found. Only in modified breakfast an increase in total fiber intake was found.

Table 3 shows values of clinical variables before and after breakfasts. No significant differences in anthropometric and biochemical variables were detected after the interventions.

**Table 3:** Mean values (± SEM) of clinical variables before and after Brazilian and modified breakfasts

	Brazilian			Modified		
	
	Before	After	p-value	Before	After	p-value
Body mass index (kg/m^2^)	30.2 ± 0.6	30.3 ± 0.6	0.14	30.3 ± 0.6	30.2 ± 0.6	0.23
Waist circumference (cm)	99.8 ± 1.3	99.6 ± 1.4	0.54	100.2 ± 1.3	99.7 ± 1.40	0.11
Systolic blood pressure (mmHg)	124.6 ± 2.5	123.4 ± 2.0	0.57	126.5 ± 2.3	123.5 ± 2.2	0.05
Diastolic blood pressure (mmHg)	75.1 ± 1.40	73.8 ± 1.8	0.24	75.8 ± 1.5	73.3 ± 1.5	0.02
Fasting plasma glucose (mg/dL)	95.6 ± 1.1	96.2 ± 1.1	0.42	95.3 ± 1.6	95.7 ± 1.3	0.78
Fasting insulin (µUI/mL)	40.2 ± 9.0	40.5 ± 5.5	0.98	40.1 ± 4.9	52.4 ± 5.5	0.09
HOMA-IR	8.9 ± 2.0	9.2 ± 1.2	0.89	8.9 ± 1.1	11.6 ± 1.4	0.11
Total cholesterol (mg/dL)	206.0 ± 5.7	207.9 ± 5.6	0.52	205.6 ± 5.8	204.7 ± 5.1	0.78
HDL-c (mg/dL)	55.3 ± 1.8	53.7 ± 2.5	0.09	51.8 ± 1.8	54.2 ± 2.0	0.03
LDL-c (mg/dL)	123.6 ± 4.4	121.7 ± 4.5	0.40	121.6 ± 4.4	121.4 ± 4.0	0.94
Triglycerides (mg/dL)	149.8 ± 13.0	154.2 ± 10.5	0.56	151.2 ± 11.4	150.1 ± 11.2	0.83

After modified breakfast (Table 3), there is no variation in anthropometric measurements either; however, a significant increase in HDL-cholesterol concentration (51.8 ± 1.8 *versus* 54.2 ± 2.0 mg/dL, p = 0.03) and decrease in blood pressure levels were observed.

**Figure 2:**
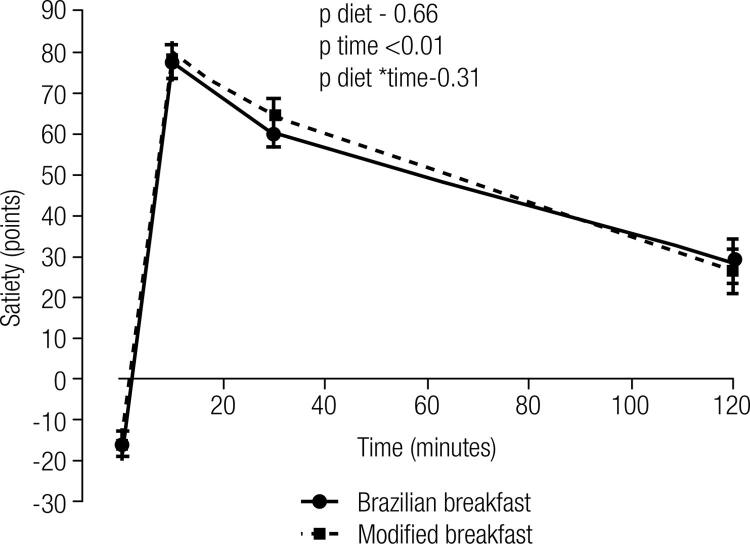
Mean values (± SEM) of the satiety scores obtained by the SLIM scale during Brazilian and modified breakfasts.

Figure 2 depicts mean values of satiety, before and after each breakfast period, showing similar profiles of the curves obtained.

Mean values of the satiety scores of participants, obtained following Brazilian and modified breakfast, are in Table 4. At fasting, as expected, participants referred negative values, indicative of hungry. Ten minutes from the consumption of both breakfasts, a significant elevation of scores was found with further gradual decrease across time (Table 4 and Figure 2). No difference in the satiety score profiles, obtained during the 120 minutes of each breakfast, was observed.

**Table 4:** Differences in score of the satiety of participants after Brazilian and modified breakfasts

	Brazilian	Modified
Fasting	-16.1 ± 3.1	-16.0 ± 3.1
10 minutes	76.5 ± 2.8	78.6 ± 3.2
30 minutes	59.7 ± 3.1	64.5 ± 3.5
120 minutes	28.6 ± 5.4	26.3 ± 5.1
Global analysis		
Effect of diet	0.66	
Effect of time	< 0.001	
Interaction diet * time	0.31	

Repeated measures ANOVA.

Associations of late satiety (30 e 120 minutes) with glicemia, insulinemia e HOMA-IR were tested, after breakfasts, but no significant correlation was detected (data not shown).

## DISCUSSION

In clinical practice, the availability of a handy tool to evaluate dietary interventions on satiety of overweight individuals is of great interest. Applying the SLIM scale, this study compared the satiety perception exhibited by distinct compositions of breakfasts to which there is evidence of deleterious or beneficial effects on the cardiometabolic profile. Isocaloric breakfasts were contrasting according to the type of fatty acids and fiber contents; the modified one showed components of the Mediterranean diet. Using this scale that quantifies satiety level, our findings did not support the hypothesis that part of the effect of these diets might occur via satiety control. Despite the benefits of the modified breakfast (blood pressure reduction and HDL-cholesterol elevation), the negative result regarding its association with satiety could indicate the limitation of the tool in detecting changes on an ample and complex regulation system of the energy balance. In addition, it is possible that a high-fat diet, independently of the fatty acids proportion, causes similar impacts in satiety perception.

The participants showed a mean body mass index compatible with class I obesity and increased waist circumference in both sexes, characteristics that make them susceptible to metabolic syndrome. Since there was no counseling for food restrictions or physical activities, substantial changes in anthropometry were not expected, despite two previous meta-analysis had shown significant Mediterranean diet-induced changes in waist circumference (26,27). The lack of anthropometric changes in our study reinforces at some extent that modification in a single meal like our intervention is unable to change satiety perception to a level that would impact in body adiposity.

Reduction in blood pressure levels induced by a Mediterranean-style diet was already described (26,28). Interestingly, we detected a decline in blood pressure after modification in a single meal for 4 weeks. Evidence obtained in an epidemiological study did not support that antihypertensive effects of Mediterranean diet would be attributed to whole grains (29) but to vegetables – due to their potassium content – and/or to the olive oil (30), which were used in our modified breakfast. Based on animal studies, it is speculated that antioxidant actions of oleic acid-rich oils (such as extra-virgin olive oil) contribute to the improvement in blood pressure levels (31).

Noteworthy that both breakfasts promote high level of satiety; in other studies, when foods were consumed mainly at dinner, the self-reported satiety was close to the scores obtained in our study (20). For both sex and after both breakfasts, mean values of satiety scores were slightly higher in women than men, and reached significance 10 minutes after the modified breakfast. Such possible difference in this perception deserves to be examined in a bigger sample. Investigations on the effects of macronutrients on satiety have shown that fat has less satietogenic properties than carbohydrates (14,15). In current study, fat was offered to participants in relatively high proportions, with predominance of SFA in the Brazilian breakfast and unsaturated fatty acids in the modified one. High MUFA and PUFA contents in the later were achieved due to the presence of olive oil and peanuts, respectively. Deleterious effects of SFA in hypothalamic pathways of body weight control were described in animal models (32). High-saturated fat diet-fed mice exhibited inflammation in hypothalamus and reduced orexigenic signals, resulting in weight gain and insulin resistance. Our findings of comparable satiety levels, following periods of exposure to distinct breakfasts for 4 weeks, do not corroborate the hypothesis that subtypes of fatty acids would induce distinct effects in central mechanisms which modulate food intake and satiety. To our knowledge, there is no similar report limiting the comparison of our results with literature. However, some investigators infused emulsion with different fatty acids contents in the ileum and verified that MUFA but not SFA infusion was associated with increased satiety (18).

The higher fiber content in the modified breakfast could also influence satiety perception, considering that some studies had reported that whole-grain foods increase satiety (15,17,33), but not others (34). Facing contrasting results concerning their effect on energy intake, glucose excursion and satiety, it was desirable to expand knowledge on this aspect. The fiber content in the modified breakfast resulted in significant increase in daily consumption of fibers. Previous studies that evaluated glycemic index were also controversial regarding the inverse correlation of this parameter to satiety (35,36). We did not detect correlation between the satiety scores and levels of plasma glucose nor insulin. It is possible that more substantial modifications in the meals could reach results different from ours. Also, we cannot exclude that the participants were already used to the Brazilian pattern, therefore maintaining their satiety level when this breakfast was consumed.

The modified breakfast could have provoked reductions in plasma glucose, insulin and HOMA-IR. Unsaturated fatty acids in animal models and *in vitro* studies improve insulin signaling inducing glucose uptake (37). The effects of olive oil, due to its MUFA content, on attenuation of inflammatory status and insulin resistance are well documented (38,39), and this ingredient has been recommended for prevention and control of cardiometabolic diseases (26,40). The lack of HOMA-IR improvement in this study may indicate that modification of a single meal is insufficient to benefit glucose metabolism. High fiber intake has been also associated with cardioprotection mainly due to its beneficial effects on lipid metabolism (41,42). Actually, only the modified breakfast induced improvement in lipid profile reflected by the elevation in HDL-cholesterol concentrations, which should be maximized due to the consumption of olive oil (43).

The reduced sample size may express difficulties of individuals to sustain lifestyle changes in real life. Despite subjectivity of satiety and intrinsic limitation of the tools to assess this perception, our findings may suggest that some of the recognized benefits of the Mediterranean diet, that should affect hormonal and neural pathways, have low impact on satiety and maybe food intake.

We conclude that isocaloric breakfasts that differ according to fatty acids content are unable to alter satiety levels, which are not correlated to glucose parameters in overweight individuals. Stronger modifications of daily meals might be necessary to differentiate satiety levels under distinct dietary patterns.
